# Study of patterned GaAsSbN nanowires using sigmoidal model

**DOI:** 10.1038/s41598-021-83973-9

**Published:** 2021-02-25

**Authors:** Sean Johnson, Rabin Pokharel, Michael Lowe, Hirandeep Kuchoor, Surya Nalamati, Klinton Davis, Hemali Rathnayake, Shanthi Iyer

**Affiliations:** 1 Department of Electrical and Computer Engineering, North Carolina A&T State University, Greensboro, NC 27411 USA; 2 Nanoengineering, Joint School of Nanoscience and Nanoengineering, North Carolina A&T State University, Greensboro, NC 27401 USA; 3 Nanoscience, Joint School of Nanoscience and Nanoengineering, University of North Carolina At Greensboro, Greensboro, NC 27401 USA

**Keywords:** Electrical and electronic engineering, Nanoscale materials, Nanowires, Nanowires

## Abstract

This study presents the first report on patterned nanowires (NWs) of dilute nitride GaAsSbN on p-Si (111) substrates by self-catalyzed plasma-assisted molecular beam epitaxy. Patterned NW array with GaAsSbN of Sb composition of 3% as a stem provided the best yield of vertical NWs. Large bandgap tuning of ~ 75 meV, as ascertained from 4 K photoluminescence (PL), over a pitch length variation of 200–1200 nm has been demonstrated. Pitch-dependent axial and radial growth rates show a logistic sigmoidal growth trend different from those commonly observed in other patterned non-nitride III–V NWs. The sigmoidal fitting provides further insight into the PL spectral shift arising from differences in Sb and N incorporation from pitch induced variation in secondary fluxes. Results indicate that sigmoidal fitting can be a potent tool for designing patterned NW arrays of optimal pitch length for dilute nitrides and other highly mismatched alloys and heterostructures.

## Introduction

Nanowires (NWs), due to their unique properties arising from one-dimensional (1D) architecture, demonstrate excellent enhancement of light confinement, photosensitivity, and optical absorption for optoelectronic applications such as photodetectors, optical switches, optical interconnects, and solar cells^1–7^. Additionally, NWs grown via selective area growth (SAG) techniques, which is a hybrid of top-down and bottom-up nanofabrication methods, further enhance these properties by patterning the substrate to precisely position nucleation sites for self-assisted vapor–liquid-solid (SA-VLS) growth of vertical NWs. This enables scalability and exploiting the effects of pattern designs for bandgap tuning while acting as waveguides for impinging light leading to higher absorption and responsivity^8–12^.

III-V semiconductor alloys are the primary source materials for these applications. Amongst the various alloys, GaAsSb is an important material system covering the telecom wavelength range from 870 nm (GaAs) to 1700 nm (GaSb). Additionally, the incorporation of a small amount of nitrogen, typically less than 2%, in the host lattice leads to a giant reduction in the bandgap as well as the lattice constant, which is unique to the dilute nitride alloy system. This enables bandgap tuning over a much wider range and a broader selection of heterogeneous material integration. Furthermore, the distinguishing characteristics of the dilute nitride GaAsSbN material system are the independent tuning of the conduction band and valence band offsets by N and Sb, respectively. This, when combined with the reduction of the lattice parameter, provides additional flexibility in controlling the stress–strain management with lower lattice parameter mismatched substrates, such as GaAs and Si, which are critical for device applications^13–18^.

Much work^13,14,19–24^ has been done on the study of this material system in thin films and its applications. Braza et al.^25^ reports on Sb and N interplay in GaAsSbN alloys latticed matched to GaAs with an emission range of ~ 1.0–1.16 eV for solar cells and photodetector applications. Xu et al.^26^ demonstrates the applicability of GaAsSbN as the absorption layer in a GaAsSbN/GaAs PIN waveguide photodetector with DC responsivity of 0.44 A/W.

Despite the potential benefits of dilute nitride NW compounds for optoelectronics, there is only limited work that has been reported on these NWs. Kasanaboina et al.^27^ reported on GaAs/GaAsSbN/GaAs core-multishell NWs with 4 K photoluminescence (PL) emission showing good homogeneity, redshift, and close lattice match as observed by X-ray peak to confirm N incorporation. Also, Kasanaboina et al.^28^ reported on the effect of N_2_ ambient *ex-situ* annealing on core-multishell NWs. Their results demonstrated the annihilation of N-induced defects evidenced by the lower energy PL peak corresponding to band tail states, vanishing on post-annealing. Sharma et al.^29^ report bandgap reduction by 10% for defect-free axial dilute nitride GaAsSbN NWs along with enhanced N incorporation via N_2_ ambient in-situ annealing, as evidenced by the PL spectra. Deshmukh et al.^19^ demonstrates high-density ternary GaAsSb core-multishell GaAsSb/GaAsSbN/GaAlAs NWs with a reported redshift of ~ 160 meV in comparison to a non-nitride shell. These previous reports demonstrate the applicability of the GaAsSbN material system for non-patterned NWs. There have been no reports of vertical GaAsSbN NWs grown on patterned substrates.

The patterning of GaAs and GaAsSb NW arrays have been reported to have tuning effects on the bandgap^30^ and, therefore, on the optical response of the photodetector. It is to be noted that pattern design has an effect on the pre-deposition parameter, and the axial and radial growth rates of the NWs^10,31,32^. Furthermore, the growth of vertical NWs by SA-VLS depends on the interdependent processing and growth parameters^9,33^.

In this work, the first reports on patterned NWs of GaAsSbN grown SA-VLS mechanism by plasma-assisted molecular beam epitaxy (MBE) on Si (111) substrates are presented. Pitch dependent growth studies are investigated and analyzed in comparison to non-nitride patterned NW samples and modeling techniques. Pitch-dependent growth models developed by Gibson et al.^31^ and Sharma et al.^10^ for non-nitride GaAs and GaAsSb patterned ensemble NWs were found to be inadequate to describe the pitch-induced axial and radial growth rate variations completely. Hence, a different modeling approach, namely a logistic growth sigmoidal model, which is used more often in biological, geological, and chemical sciences where the binding of one atom affects subsequent atoms cooperatively^34–36^, was investigated. There has been limited use of sigmoidal modeling in semiconductor heterointerfaces, demonstrated by Luna et al.^37^, wherein the growth and interface process has been reported to form a cooperative system. Lu et al.^38^ further extended this technique to the modeling of Sb at. % in InAsSb-InAs heterointerface grown by MBE. Munoz et al.^39^ demonstrated the sigmoidal fitting method in the RF sputtering of thin films.

This is the first report of using the sigmoidal function to model axial and radial growths in patterned NWs. We have shown that the extracted parameters from the sigmoidal model provide deeper insight into the pitch-dependent interplay between Sb and N, which correlated well with the variation in PL spectral peaks. Pitch-dependent bandgap tuning of ~ 75 meV for a pitch length variation of 200 nm to 1200 nm is demonstrated.

## Experimental details

The p-type Si(111) substrate sample with a SiO_2_ layer of 15 nm was patterned with square arrays (50 × 50) of patterned holes via Elionix ELS-7500 EX Electron Beam Lithography System^10,33^. The sample is spin-coated with PMMA at 3000 rpm for 40 s and soft-baked at 180 °C for 120 s. Patterned hole diameter for the samples was 200 nm. Pitch length varied between 200 and 1200 nm.

GaAsSbN NWs are grown on the patterned substrates at a growth temperature of 620 °C by plasma-assisted molecular beam epitaxy via Ga-assisted VLS growth mechanism using the EPI 930 solid source MBE system, which consists of valve-controlled As and Sb crackers, and radio-frequency (RF) controlled N-plasma source. Two samples of the patterned arrays, A and B, are the focus of this study. Sample A refers to the configuration of GaAs followed by GaAsSb composition of 7% Sb composition grown for segment lengths of ~ 600 nm at 620 °C and ~ 700 nm at 590 °C, respectively. GaAsSbN segment was then grown axially for a segment length of ~ 1800 nm with the nitride growth plasma configured at 300 W RF. Sample B refers to the configuration of GaAs followed by GaAsSb composition of 3% Sb composition grown for segment lengths of ~ 225 nm and ~ 800 nm at 620 °C and at 590 °C, respectively, with the final GaAsSbN segment grown for a segment length of ~ 1000 nm at 7% Sb. The beam equivalent pressure (BEP) for the As_4_ flux was 4.8 × 10^–6^ Torr for the GaAs stem and 3.6 × 10^–6^ Torr for axial GaAsSb growth with N BEP of 1.8 × 10^–7^ Torr for dilute nitride incorporation. The BEP for the Sb flux was 8.6 × 10^–7^ Torr for both the GaAsSb stem and axial GaAsSbN growth, unless otherwise noted. The Ga cell temperature with As cracker cell temperature configured for As_4_ species was preset to a V/III ratio of 20 to yield an equivalent GaAs thin film growth rate of 0.5 monolayer/second. The summary of the growth durations of sample A and B is presented in Table 1.

**Table 1:** Summary of the growth duration and approximate axial segment length for samples A and B.

	Sample A		Sample B	
	Axial length		Axial length	
GaAs stem	600 nm		225 nm	
GaAsSb stem	700 nm		800 nm	
GaAsSbN	1800 nm		1000 nm	
Total	~ 3100 nm	17 min	~ 2025 nm	10 min

Morphological characterization of the patterned NWs was accomplished by the Carl Zeiss Auriga-BU FIB field emission scanning electron microscope (FESEM). JEOL 2100PLUS high-resolution transmission electron microscope (TEM) was used for transmission electron images, selected area electron diffraction (SAED) patterns, and energy dispersive X-ray spectroscopy (EDS) patterns. Optical characteristics of the NWs were captured via micro-photoluminescence (µ-PL) at room temperature (RT) and at 4 K using a Montana Cryostation and a Horiba Jobin Yvon confocal microscope with a 633 nm He–Ne laser excitation source. Axial and radial growth modeling was performed using Matlab and Origin softwares.

## Results and discussions

**Figure 1:**
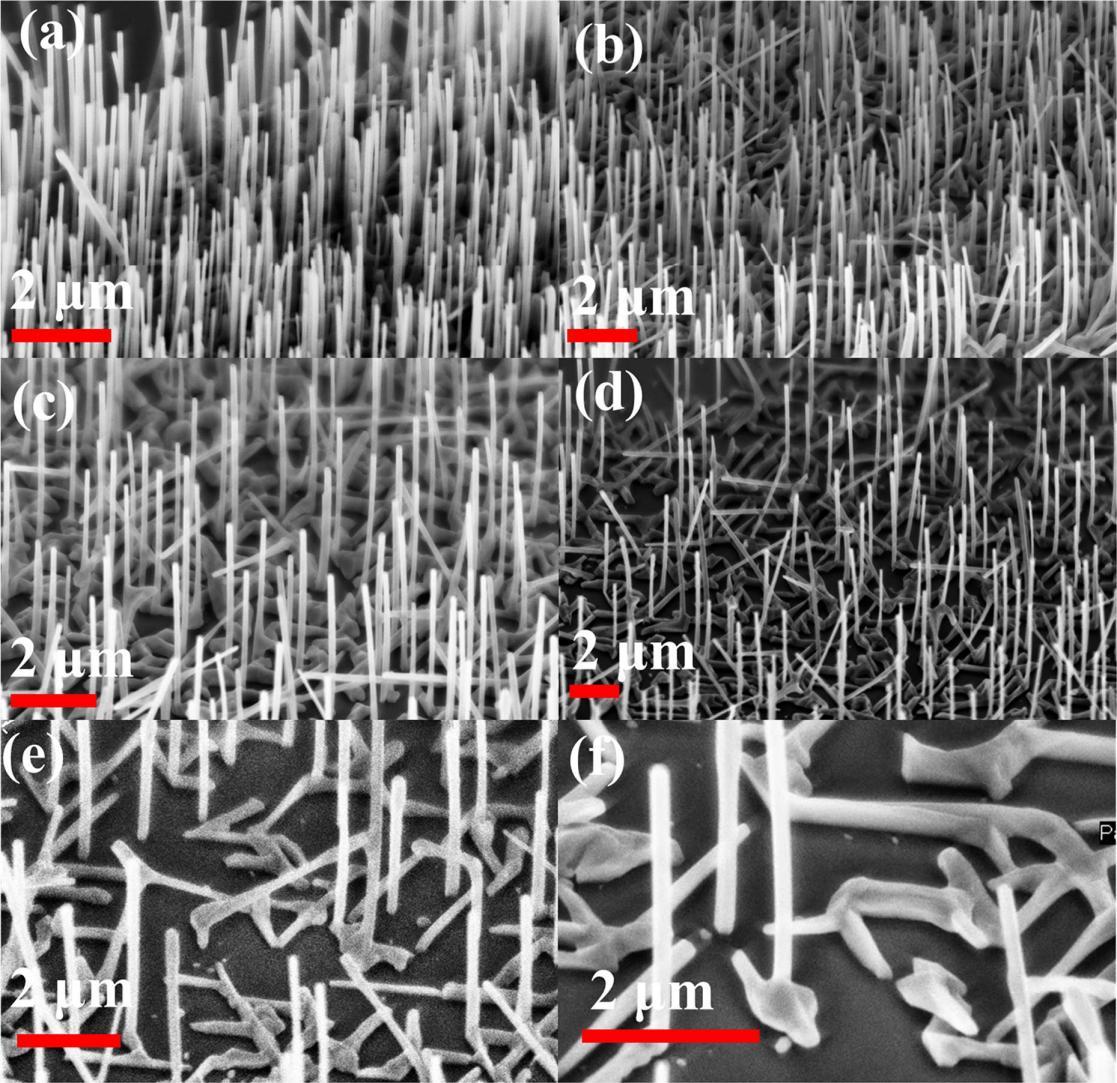
SEM images for GaAsSbN sample A for pitch lengths of (**a**–**f**) 200, 400, 600, 800, 1000, and 1200 nm.

The optimization of the growth process identifies that hole diameter, oxide height, an etching process, and pre-deposition time are some of the factors that chiefly affect vertical yield^8,9,33,40^. As nitride NWs showed much less NW density than non-nitride NWs^29^ grown under similar growth conditions on non-patterned substrates, the optimization parameters will also be affected for the vertical yield at each pitch on patterned samples. For NWs grown on patterned substrates with nitrogen in the growth environment, a diameter of 200 nm was found to provide the best results for a Ga opening time of 20 s in the two sample results presented. The NW growth consists of a GaAs stem, GaAsSb stem, and then the GaAsSbN NW. The first sample, sample A, showed the best vertical yield occurs at the pitch values of 200 nm and 400 nm, as in Fig. 1a,b. As the pitch increases, there is an increase in bent and horizontal NWs growing out of the patterned holes, as shown in Fig. 1c–f. 2D growth and random NW nucleation evident in non-patterned samples are not present in nitride NW patterned samples by comparison (Supporting Information, Figure S1).

**Figure 2:**
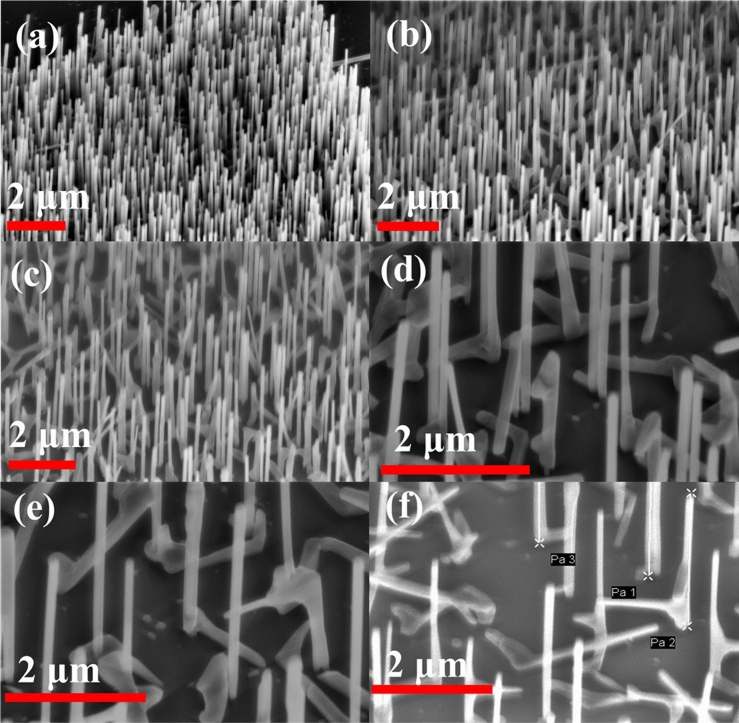
SEM images for GaAsSbN sample B with growth time of 10 min for pitch lengths of (**a**–**f**) 200, 400, 600, 800, 1000, and 1200 nm.

The presence of bent and horizontal NWs we attribute to strain likely due to compositional gradients and high Sb content^41^. To verify this supposition, the second sample, sample B, was grown under the same conditions, but with a reduced Sb flux (BEP of 1.2 × 10^–7^ Torr) for the GaAsSb stem and reduced growth time from 17 min down to 10 min. A significant increase in vertical yield was observed at each pitch as a result of the modified conditions, as seen in Fig. 2a–f. Multiple NWs are observed growing out of a single patterned hole for pitch lengths between 200 and 800 nm, which results in some coalescing of NWs and non-vertical NWs growing from patterned holes along with vertical NWs.

**Figure 3:**
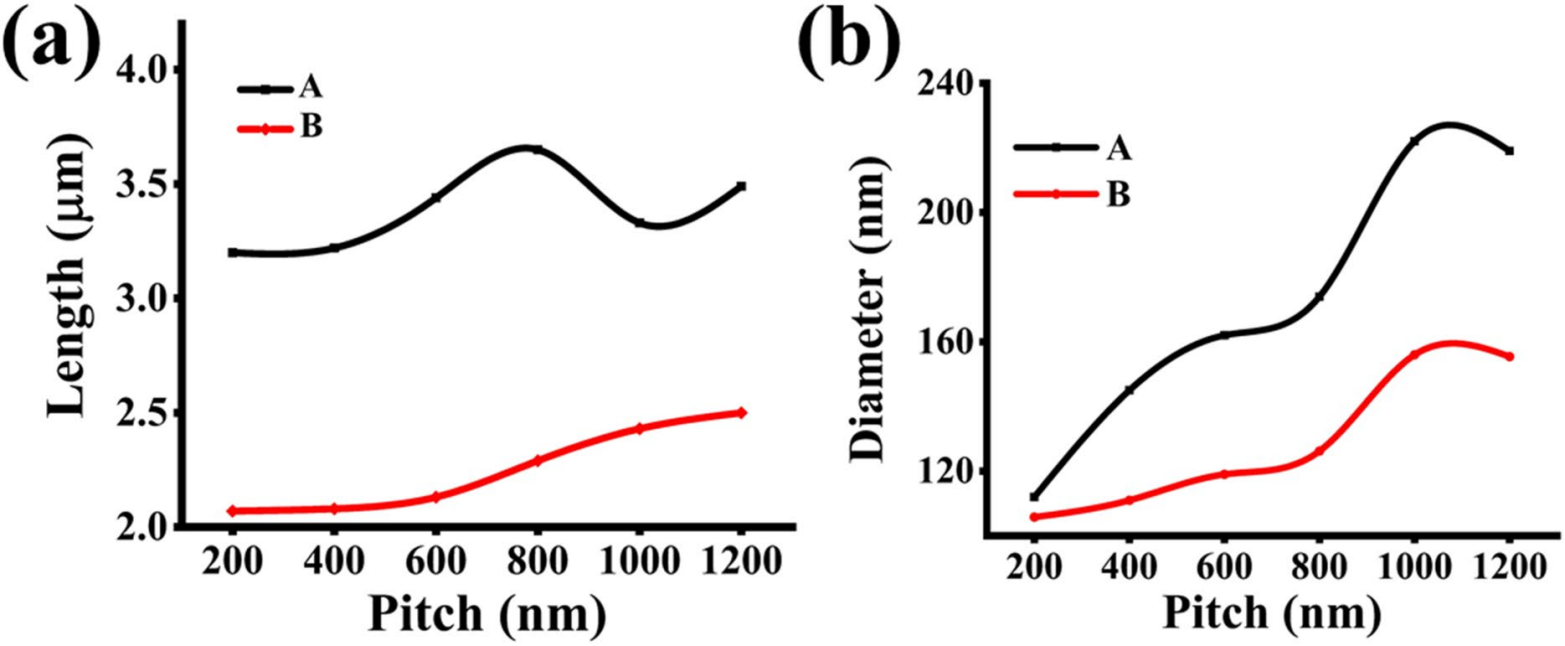
(**a**) Length versus pitch for high vertical yield samples. (**b**) Diameter versus pitch for high vertical yield samples.

In comparison with the results of non-nitride GaAsSb patterned NWs reported by Sharma et al.^10^, variations in lengths are less pronounced for smaller pitches GaAsSbN NWs, as shown in Fig. 3a. Generally, both samples are exhibiting an increase in axial growth rate with the pitch length from 400 nm, though a decrease in the axial growth rate is observed for higher pitches in sample A.

The radial growth rate was found to increase with pitch length for both samples up to a pitch length of 1000 nm, and thereafter it plateaus, as inferred from the diameter variation of the NWs in Fig. 3b.

**Figure 4:**
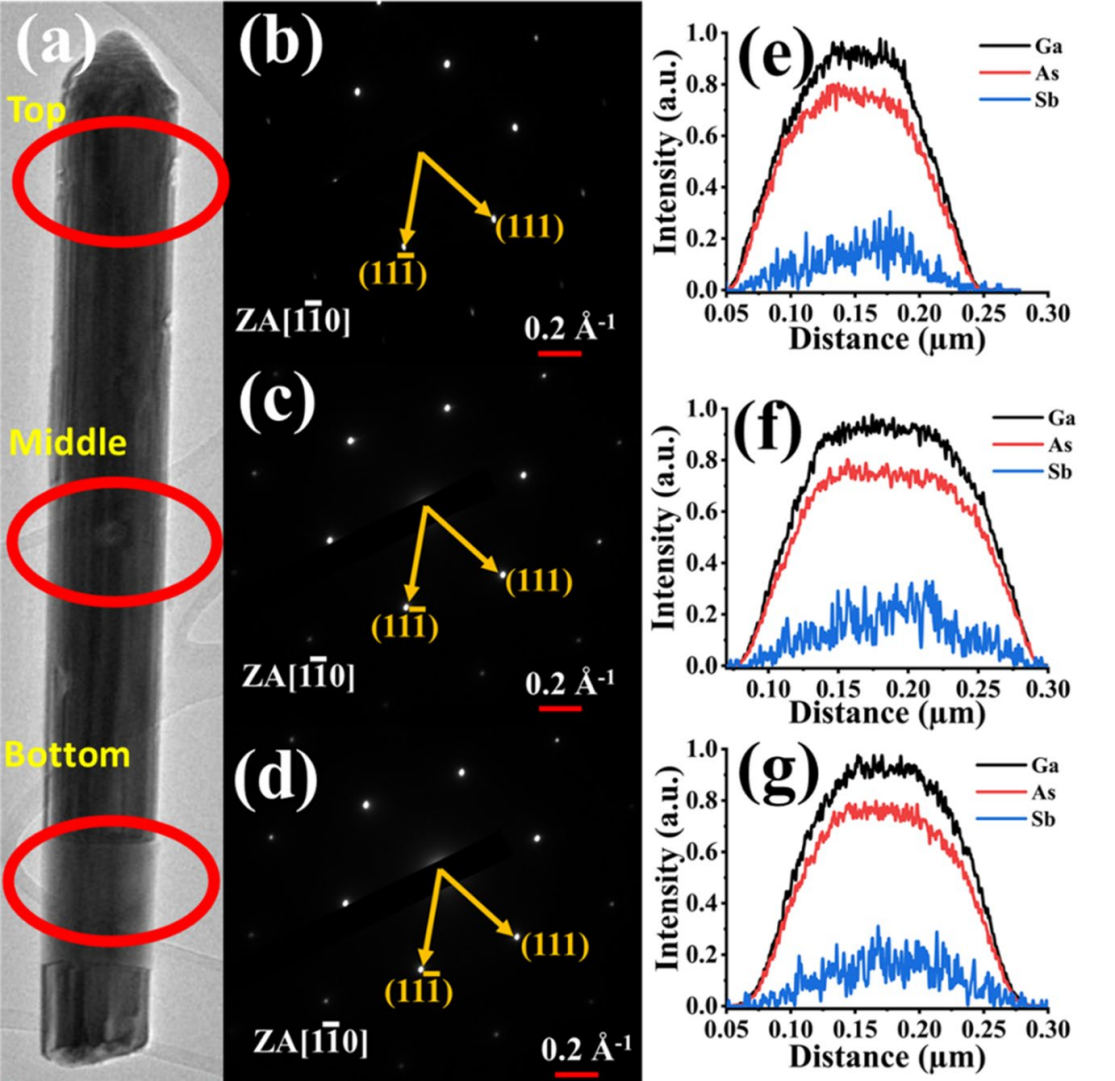
(**a**) Bright-field TEM image for dilute nitride NW, (**b**–**d**) selected area electron diffraction (SAED) for the top, middle, and bottom segments of the NW, and (**e**–**g**) energy dispersive X-ray spectroscopy for the corresponding NW segments.

Figure 4a shows the TEM image of the NW from the patterned growth. Top, middle, and bottom NW segments captured via SAED characterization confirmed the single-crystal zincblende structure of the NWs, as seen Fig. 4b–d. The absence of satellite spots in the SAED pattern indicates the NWs are free of planar defects. EDS measurements, shown in Fig. 4e–g, verified the 7% Sb composition for the growth design and showed compositional uniformity through the bottom and middle of the NW. Among the three constituent elements Ga, As, and Sb, Sb exhibits non-uniform composition variation radially, mainly at the mid and the top segments of the NW. This is attributed to the reduction in the N-Sb exchange mechanism with the NW growth compared to the bottom section due to the less duration of exposure of N, hence not sufficient time to reach the steady-state. The composition of N is too low to be detected within these NWs.

### Pitch-dependent PL spectral response

**Figure 5:**
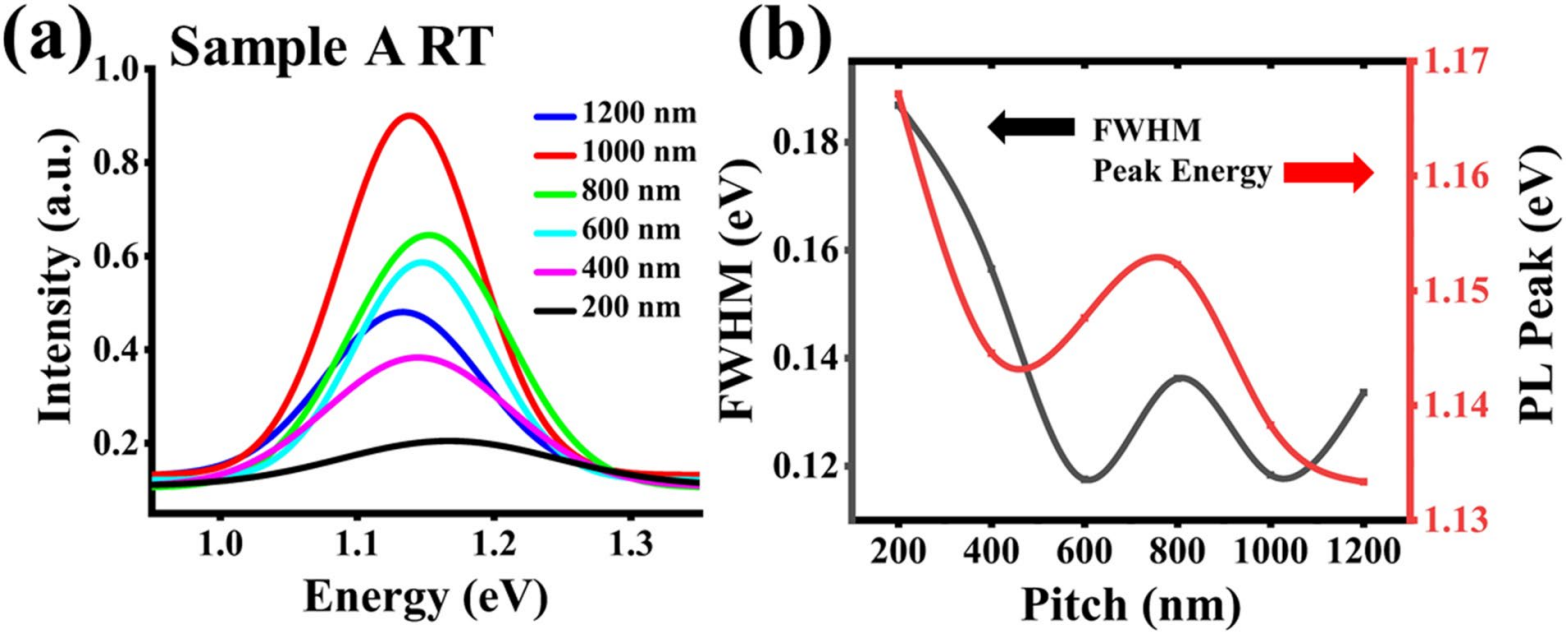
Sample A: (**a**) Gaussian fit for RT PL measurements, and (**b**) full-width-half-maxima and Gaussian energy peaks versus pitch.

Next, we examined the pitch dependency of the PL characteristics. Figure 5a shows the RT spectra of sample A of different pitches. At RT, a 34 meV redshift in PL was observed for the pitch length increase from 200 to 1200 nm.

In Fig. 5b, the pitch variations of PL full-width-half-maxima (FWHM) and peak energy results are shown. The FWHM, a relative measure of the quality of the NWs at each pitch, shows a decrease from 200 to 600 nm pitch lengths; thereafter, it oscillates from 600 to 1200 nm. Furthermore, the peak energy plot of Fig. 5b shows the overall redshift in PL energy, as mentioned previously, with a small blue shift between the 400 nm and 800 nm pitch lengths.

**Figure 6:**
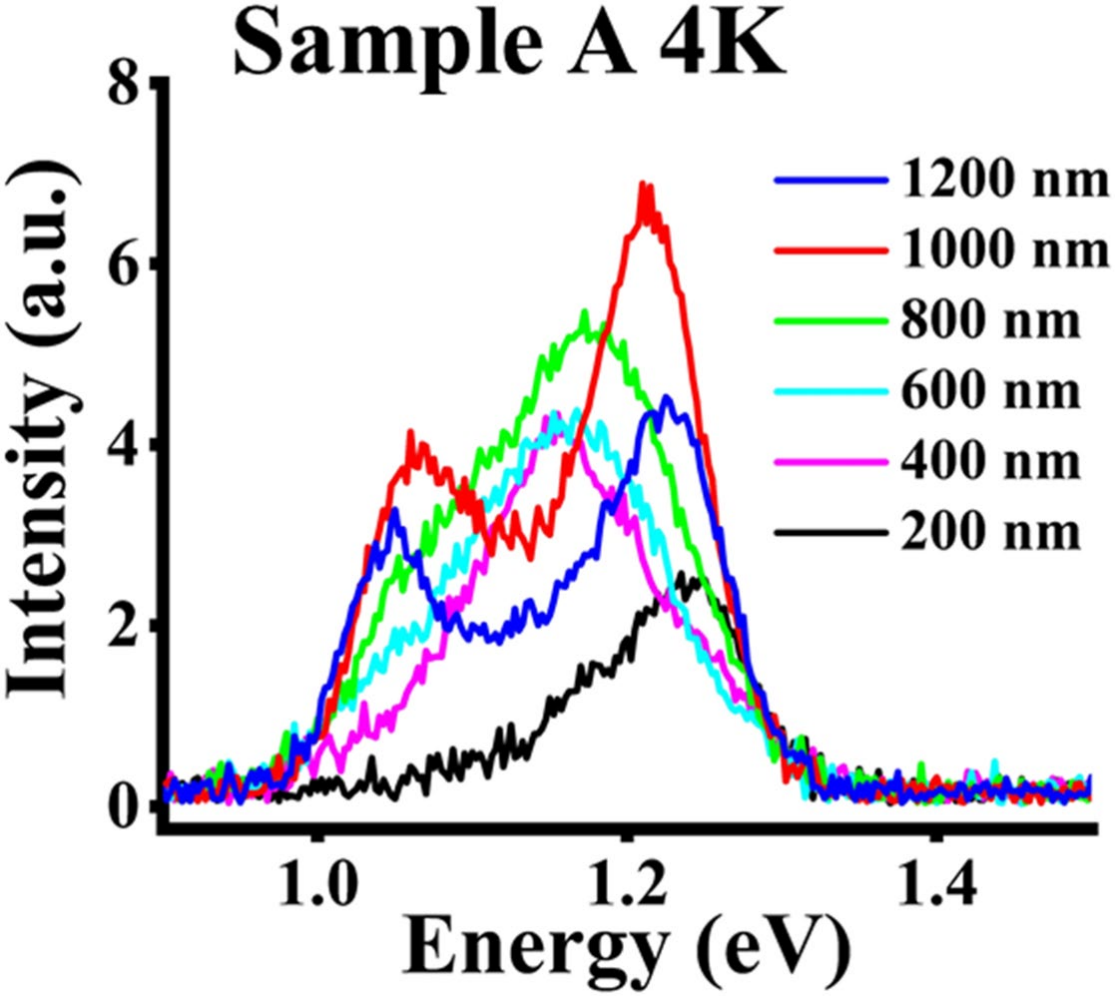
4 K PL measurements for sample A for different pitch lengths.

Exhibited in Fig. 6 are the 4 K PL results for sample A. The 200 nm PL characteristic exhibits asymmetric shape with the highest peak energy of ~ 1.23 eV amongst all the spectra. Analysis of the PL spectra reveals a low energy peak of ~ 1.16 eV of low intensity. With increasing pitch length to 600 nm, the PL responses are more symmetric and broader, revealing a redshift of 73.3 meV, which is greater than the redshifts of ~ 45 meV presented for non-nitride NWs^10^ over a much wider pitch length variation. With an increase in pitch length to 800 nm and beyond, the PL response becomes increasingly more non-symmetric. Deconvolution of the response at 800 nm reveals the presence of the second peak of lower intensity and lower energy at ~ 1.07 eV, which indicates N incorporation and becomes distinct in NWs grown at higher pitch lengths. It is observed that the high energy PL peaks of 1.21 eV and 1.22 eV for the 1000 nm and 1200 nm pitches, respectively, are very close to the 4 K peak energy observed for the NWs grown at the 200 nm pitch (1.23 eV). It is surmised that the high energy peak ~ 1.21–1.18 eV is associated with Sb peak, which is also consistent with the Sb flux used based on our earlier work^27,28,41^. A similar PL result indicating the low energy peak as a result of N incorporation was also reported by Sharma et al.^29^ for non-patterned GaAsSbN under N ambient annealing. At the 200 nm pitch length, the asymmetric shape of the PL spectra with the long tail protruding at lower energies is indicative of N related band tail states. Additionally, the presence of the dominant PL peak at a higher energy for the 200 nm pitch length is representative of GaAsSb rich composition with very little N incorporation in the host lattice^28^. Moreover, the broad peaks and symmetric PL spectra observed at pitch lengths of 400 nm and 600 nm suggest an increase in N incorporation as well as a more homogeneous incorporation of Sb and N within the composition of the NWs.

**Figure 7:**
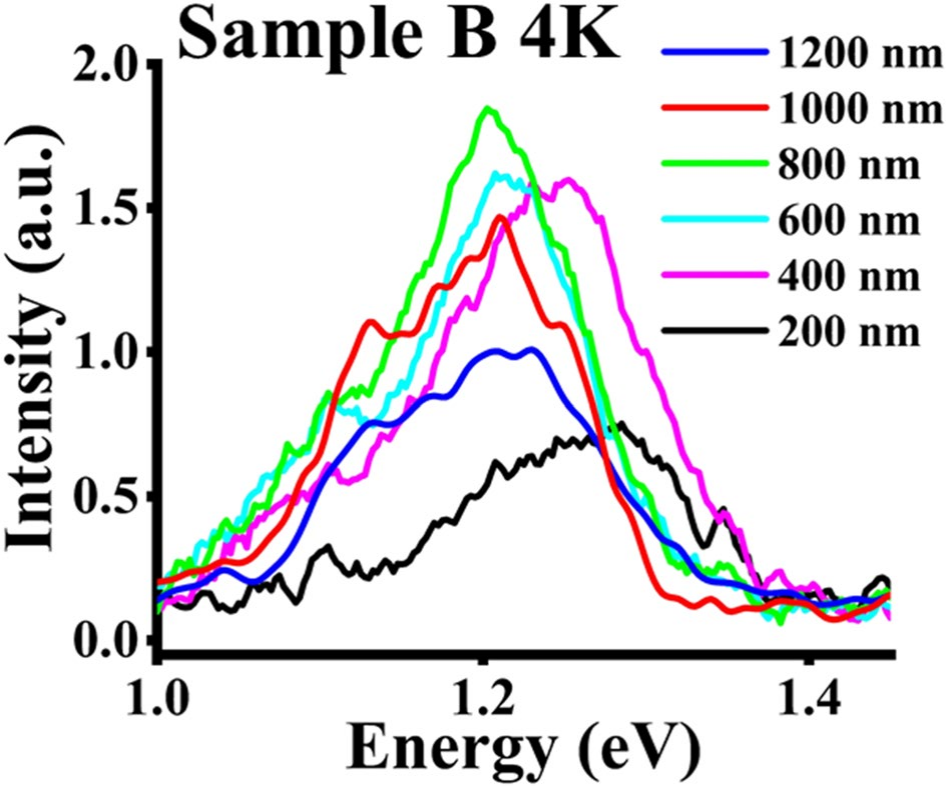
4 K PL measurements for sample B for different pitch lengths.

For sample B, an RT PL peak was not observed. The 4 K PL results are depicted in Fig. 7. Two PL peaks are distinguishable in the 4 K PL measurements of sample B for each pitch length. The deconvolution of the 4 K PL measurements reveals a distinguishable low-intensity peak of 1.10 eV at 200 nm pitch length red shifting to 1.09 eV at 800 nm. Both the higher and lower energy peaks redshift by ~ 73 meV and 12 meV, respectively, until the 1000 nm pitch length. It is worth noting that the low energy peak associated with N becomes discernible at the pitch lengths of 400 nm and beyond (Supporting Information, Figure S2), as in sample A. However, for pitch lengths of 1000 nm and 1200 nm, there is a decrease in the overall PL intensity with broadening of the overall spectra, which contrasts with the more distinct N peaks in sample A. Furthermore, in both the cases at a lower pitch of 200 nm, the higher energy peak is dominant, indicating the presence of primarily GaAsSb composition and negligible incorporation of N. Additionally, a reduction of ~ 10% from the high energy to low energy PL peaks is observed, which is consistent with the PL spectra reported for N incorporation in non-patterned GaAsSbN NWs^29^. A summary of the pitch-dependent PL results is provided in Table 2.

**Table 2:** Summary of patterned 4 K spectral response results.

Pitch length (nm)	Sample A			Sample B		
	High energy peak (eV)	Low energy peak (eV)	LE/HE ratio (%)	High energy peak (eV)	Low energy peak (eV)	LE/HE ratio (%)
200	1.23	1.16	94	1.26	1.10	86
400	1.16	1.00	86	1.24	1.10	89
600	1.15	1.04	90	1.22	1.09	90
800	1.18	1.07	90	1.21	1.09	90
1000	1.21	1.08	89	1.22	1.13	93
1200	1.22	1.06	87	1.21	1.11	92

## Axial and radial growth in patterned ensemble nanowires

Direct impinging flux and secondary fluxes each contribute to the growth rate in the VLS mechanism, as identified by Gibson et al.^31^ Sharma et al.^10^ identified that axial and radial growth rates depend on (i) effective beam area intercepted by the droplet or NW sidewall facets, (ii) shadowing of the direct impinging flux, (iii) shadowing of the line of sight re-emitted flux, and (iv) diffusion lengths of the growth species on the substrate and NW sidewall facets. The secondary fluxes identified were re-emitted flux from the oxide and re-emitted flux from the sidewall facets. It is recognized that as pitch decreases, the secondary flux from the oxide decreases, and the secondary flux from the sidewall facets increases. Supersaturation of the Ga droplet in self-assisted VLS growth is affected by the availability of group-V atoms, which directly impacts the axial growth of the NW. Furthermore, group-III atoms also affect the droplet size and the radial growth rate. Pitch plays a direct role in the availability of group-III and group-V atoms due to shadowing, the area of the oxide surface between NWs, and the distance between a neighboring droplet and sidewall facet.

### Growth rate modeling for nitride nanowires

The use of numerical modeling can give further insight into processes that are contributing to the growth dynamics of these vertical NW systems^42^. Growth dynamics for patterned NWs are typically growth technique specific for a given material systems^10,31,42–45^. For instance^43^, selective area metal–organic chemical vapor deposition (SA-MOCVD) of III-V NWs is modeled through four-volume contributions identified as (i) top surface adsorption, (ii) side facet diffusion, (iii) patterned mask diffusion, and (iv) skirt diffusion. While in the MBE growth environment, the line of sight primary and secondary fluxes are reported to dominate over sidewall and surface diffusion^31^. In the case of MBE grown patterned non-nitride vertical GaAs and GaAsSb NWs, Sharma et al.^10^ successfully presented growth rate models for the incremental changes in axial and radial extensions, which represent the combined fluxes within the growth environment of the MBE chamber and are summarized in the following expressions

1 $$\frac{dL}{dh}={\Gamma }_{a}\left(\frac{dL}{d{h}_{p}}+\frac{dL}{d{h}_{ss}}+\frac{dL}{d{h}_{sf}}\right)$$

2 $$\frac{dR}{dh}={\Gamma }_{r}\left(\frac{dR}{d{h}_{p}}+\frac{dR}{d{h}_{ss}}+\frac{dR}{d{h}_{sf}}\right)$$

where $\frac{dL}{dh}$ and $\frac{dR}{dh}$ represent the incremental change in length and radial extension with respect to the equivalent planar deposition thickness for the combined contributions of the primary flux, and the secondary fluxes scattered off the oxide surface and re-emitted from the side facets. ${\Gamma }_{a}$ and ${\Gamma }_{r}$ represent the axial and radial incorporation factors, respectively. Details on the contributions of each flux are provided in Sharma et al.^10^.

**Figure 8:**
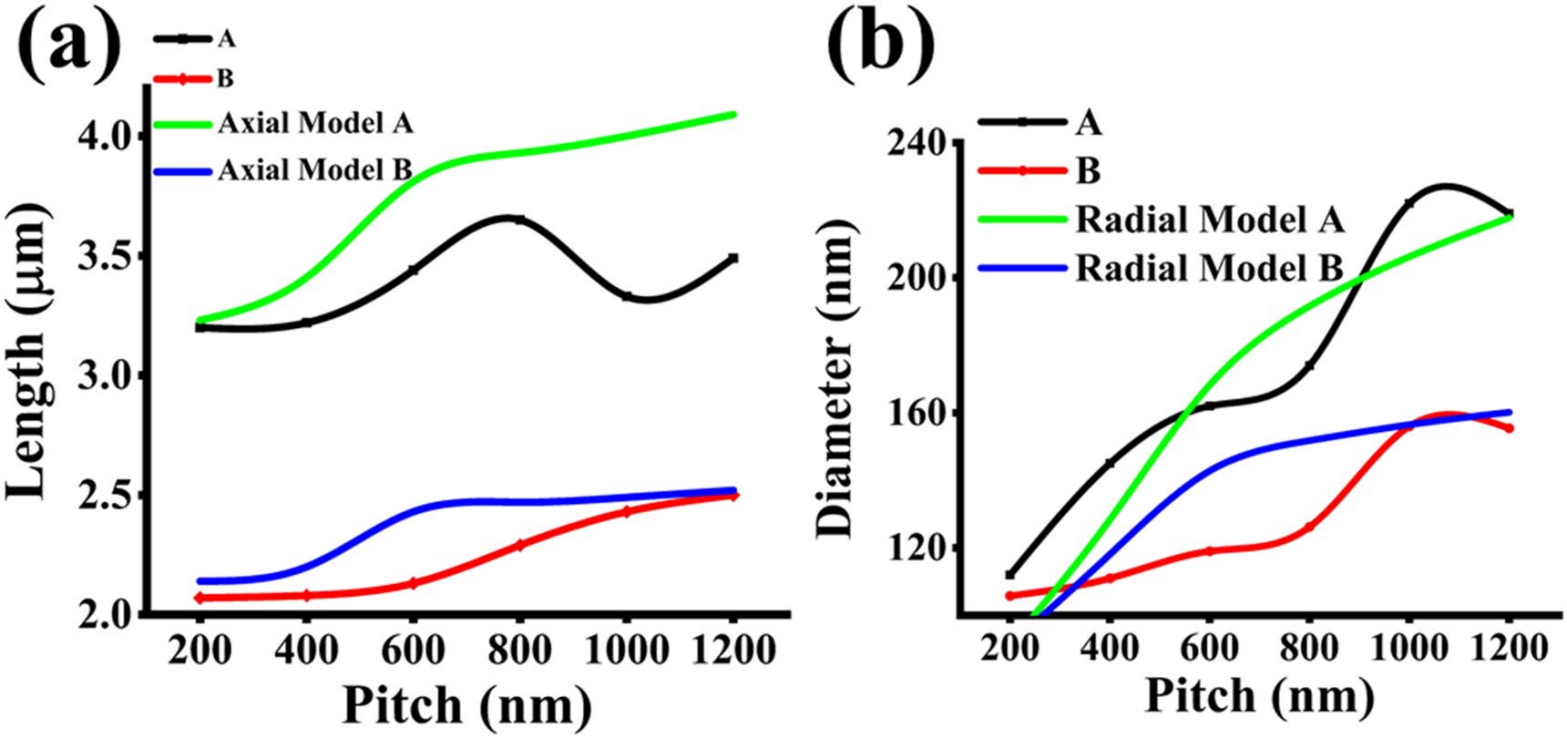
Growth rate model results for (**a**) length and (**b**) diameter in both samples A and B.

This model was implemented on the patterned nitride GaAsSbN NWs presented in this work. The variations of the length and diameter dependence on the pitch using this modeling approach do not show good agreement with the experimental data in contrast to good fitting obtained in earlier work on non-nitride NWs, as shown in Fig. 8a,b. This indicates that the model does not effectively capture the effect of the nitrogen on the axial and radial growths at each respective pitch length.

## Sigmoidal logistic growth model for axial and radial growth modeling

The growth rates of patterned nitride NWs have a combination of Sb and N induced effects as revealed by pitch dependent PL data. At the lower pitch length of 200 nm, the long tail extending at lower energies with insignificantly low N related peak intensity along with the prominent higher energy Sb related peak is indicative of Sb dominant flux. At the intermediate pitch length of 400–800 nm, the evolution of distinct N related peak affirms increased N incorporation. It has been shown that growth rates of nitride NWs are significantly lower^29^ than corresponding non-nitride NWs. Hence, the Gibson^31^ and Sharma^10^ models, which take into account the gradual changes in the growth rate with composition due to the pitch induced changes in the group V As and Sb fluxes, cannot adequately explain the variation in the nitride NWs. This calls for a different modeling approach that considers a significant change in growth rate with the compositional variation arising from the pitch length variation on the primary and secondary fluxes.

An empirical analysis approach is taken due to the observed trend within the data and further substantiated through the study of the contribution of the respective fluxes. In studying the contribution of each flux, the axial and radial growth models predict a maximum contribution from the secondary fluxes which diminish with successive time step after the nanowire reaches a particular height and diameter. This maximum contribution is predicted to occur at lower pitch lengths and is consistent with results observed in non-nitride patterned GaAs/GaAsSb NWs. However, this trend is not observed within the results obtained for patterned GaAsSbN NWs. It is surmised that the changes in the fluxes at each pitch produce average growth rates that follow a sigmoidal trend, which can be used to describe systems in where there is a transition from one steady-state to another^46–48^.

The following differential equation was formulated by Luna et al.^37^ to capture both initial nucleation and island growth in a 2D semiconductor system due to intrinsic precursor molecules and extrinsic precursor molecules, respectively, where *J* is the impingement rate and *r* is the mean growth rate.

3 $$\frac{d\theta }{dz}=\frac{J}{r}\left\{{s}_{0}\left(1-\theta \left(z\right)\right) + {s}_{1}\theta \left(z\right)\left(1-\theta \left(z\right)\right)\right\}$$

The equation has the general solution of Sigmoidal-Boltzmann form, which has been applied to the modeling of the change in material composition over an observed monolayer range.

4 $$\theta \left( z \right) = 1 - \frac{{1 + {\theta _0}}}{{1 + {e^{\left( {z - {z_0}} \right)/L}}}}$$

*θ*_*0*_ is the ratio of nucleation and island growth sticking coefficients *s*_*0*_ and *s*_*1*_, *z* is the 2D planar thickness, *z*_*0*_ is the inflection point, which shifts the *z* coordinate, and *L* represents the interface width.

In the original differential equation given by Sharma et al.^10^, the modeling is carried out in terms of incremental change in length and diameter with respect to the changes in an equivalent 2D planar deposition. The changes in the secondary fluxes, namely the scattered flux from the oxide and side facet flux, contributing towards the change in planar deposition are a function of the pitch length. Therefore, each pitch represents a change in total flux and, ultimately, a change in the deposition. In contrast, in the case of the sigmoidal model, incremental change in length is taken directly as a function of pitch. In Eq. 5, $L\left(p\right)$ means the solution of $\frac{dL}{dp}$ is described as a function of the pitch due to the effect of the secondary fluxes. We assume $\frac{dL}{dp}$, which is the change in length with respect to the change in pitch length, is of the general form of $L\left(p\right)\left(1-L\left(p\right)\right)$, which has the general solution of the Boltzmann sigmoidal function. 2D islands mediate axial nanowire growth^49^. Luna et al.^37^ postulated that 2D island growth is described by $\theta \left(1-\theta \right)$ dependence which describes the change in surface coverage for adsorbate–adsorbate interaction with respect to thin film thickness in semiconductor heterointerfaces. With the influence of pitch-dependent secondary fluxes on NW growth, a cooperative system with sigmoidal dependence is suggested.

The expression for the differential length with pitch, $\frac{dL}{dp}$, and the absolute value of length as a function of pitch, $l(p)$, is given in the following differential equation and general solution, respectively. Likewise, the sigmoidal solution is also given for diameter as a function of pitch.

5 $$\frac{dL}{dp}=L\left(p\right)\left(1-L\left(p\right)\right)$$

6 $$l\left( p \right) = {L_f} + \frac{{{L_i} - {L_f}}}{{1 + {e^{\left( {p - {p_0}} \right)/\alpha }}}}$$

7 $$d\left( p \right) = {D_f} + \frac{{{D_i} - {D_f}}}{{1 + {e^{\left( {p - {p_0}} \right)/\alpha }}}}$$

Here, *l* and *d* represent the length and diameter, respectively, *p* represents the pitch, *p*_*0*_ is the inflection point, which is the midpoint of the transition between initial and final steady-state values, and *α* describes the slope’s behavior during the transition between initial and final values.

### Sigmoidal fitting results

**Figure 9:**
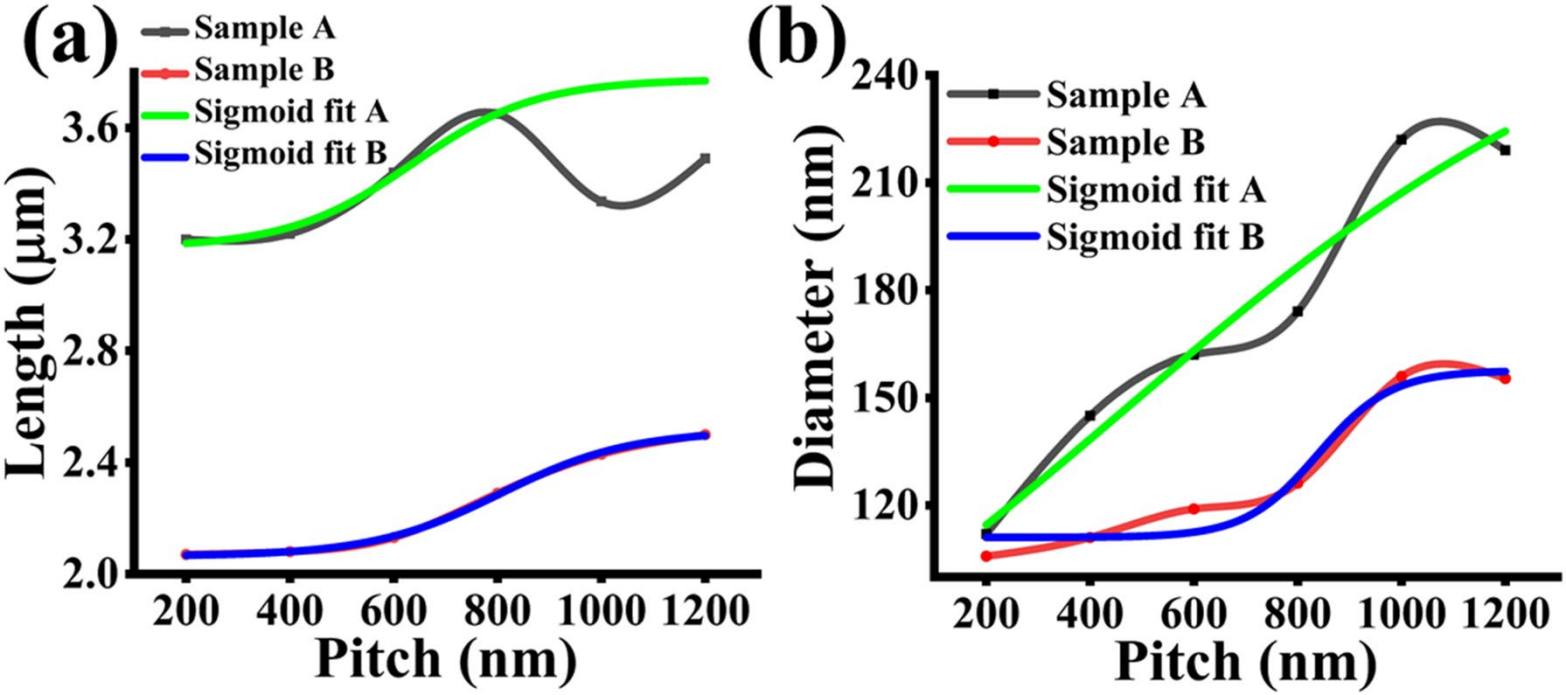
Sigmoidal fit results for (**a**) length and (**b**) diameter in both samples A and B.

The results, shown in Fig. 9a, show excellent agreement for length in sample A up through 800 nm pitch length and completely captures the growth trends for length with respect to pitch in sample B. For radial extension modeling results shown in Fig. 9b, the trend of the diameter versus pitch length is captured in sample A. The fitted model is less ‘s’ shaped and trends more closely towards a *1−e*^*(−p/α)*^ profile. The sigmoidal fitting has good agreement with the pitch dependency of the diameter in sample B. Sample A's decrease in length at the 1000 nm pitch length is very likely due to an intrinsic limitation in the NW growth caused by changes in the chemical potential of the droplet with increased growth time. Interestingly, the inflection points, *p*_*0*_*,* extracted by the sigmoidal fitting for the nitrogen samples showed good agreement with the pitch values of 600 nm and 800 nm in samples A and B, respectively, which exhibit a more broad symmetric peak encompassing both Sb and N related peak with the dominant peak being redshifted from the Sb associated high energy peak.

**Figure 10:**
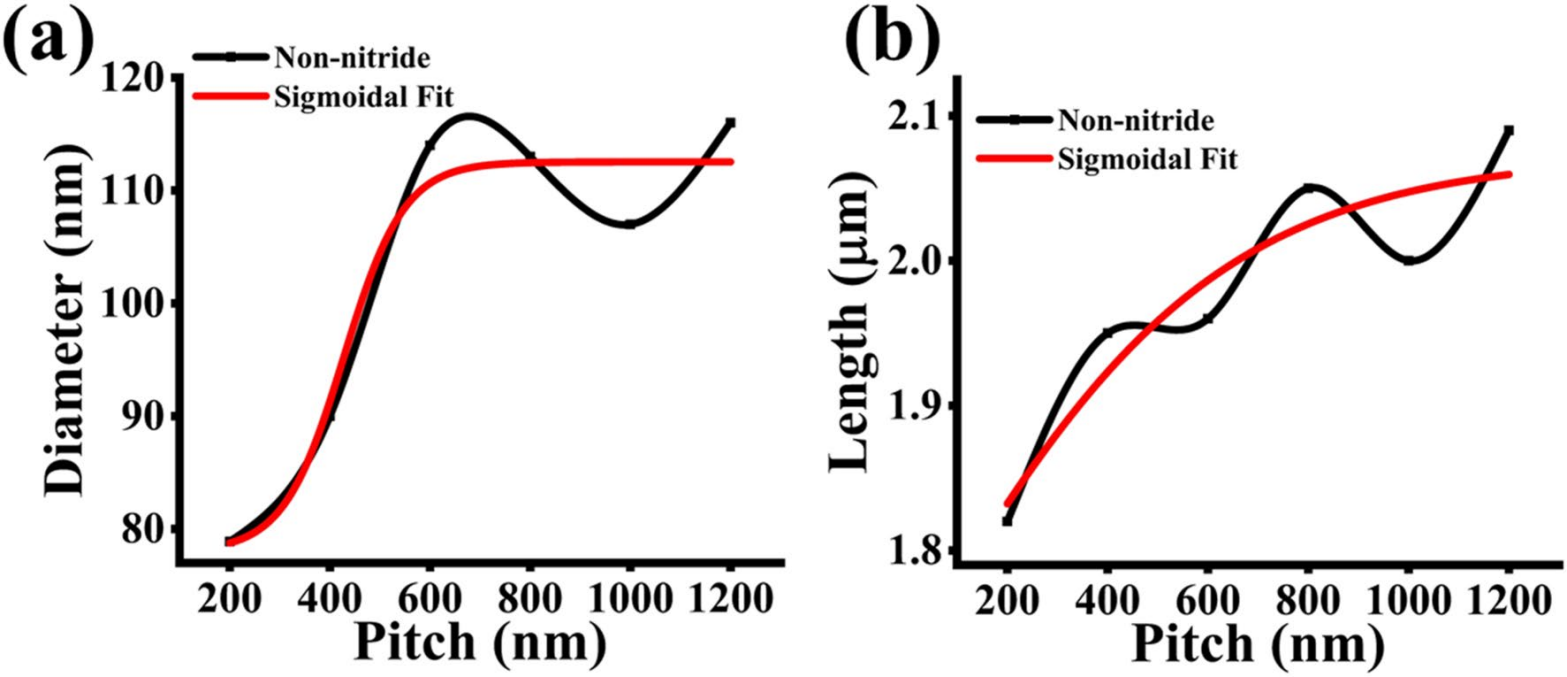
Sigmoidal fit results for (**a**) length and (**b**) diameter in the non-nitride sample.

The sigmoidal fitting was also done on non-nitride patterned axial and radial growth of Sharma’s data^10^ for comparison, as shown in Fig. 10a,b. The consistent difference in both axial and radial growth rates is in the extracted inflection point of the sigmoidal model. The data given by Sharma et al.^10^ produces a shift in inflection points toward lower pitch lengths in both cases. It must be stated that the error on the axial non-nitride inflection point is considerable. This is likely due to the minimum steady-state pitch-dependent axial growth rate occurring at a pitch length, which is lower than the available data of minimum 200 nm pitch length. A summary of the extracted parameters is given below in Table 3.

**Table 3:** Summary of sigmoidal fitting parameters for samples A, B, and non-nitride.

Parameter	Sample A		Sample B		Non-nitride	
	Axial	Radial	Axial	Radial	Axial	Radial
Initial value	3.2 µm	120	2.1 µm	111 nm	1.4 µm	78 nm
Final value	3.8 µm	273 nm	2.5 µm	158 nm	2.1 µm	113 nm
Inflection point, *p*_*0*_	636 nm	464 nm	806 nm	840 nm	13. nm	428 nm
Slope, *α*	120 nm	514 nm	124 nm	70 nm	302 nm	60 nm
Adj. R^2^	0.99	0.88	0.99	0.91	0.70	0.88

In comparison with the GaAsSb results given by Sharma et al.^10^, it is suspected that the presence of nitrogen suppresses the pitch-dependent contribution of the secondary fluxes at lower pitch lengths and shifts the inflection point to the right, which creates steady-state initial lengths and diameters at the lower pitch lengths. This suppression gives further insight into the effects of each pitch on the fluxes that contribute to axial and radial growth of nitride NWs. As the pitch increases, the contribution of the secondary fluxes takes effect and causes pitch-dependent axial and radial growths to be evidenced in the samples. It is suggested at higher pitch lengths that the values of length and diameter are due to the primary flux and the surface scattered flux dominating over the diminishing contribution of the side facet flux. Final steady-state lengths and diameters ultimately narrow down to the predominance of the primary flux contribution as the surface scattered flux continues to diminish.

In order to explain further the importance of the extracted inflection point, the first derivative of the Boltzmann sigmoidal function is calculated and is given by the following equations.

8 $$\frac{{dL}}{{dp}} = \frac{{{L_f} - {L_i}}}{\alpha }\frac{{{e^{\left( {p - {p_0}} \right)/\alpha }}}}{{{{\left( {1 + {e^{\left( {p - {p_0}} \right)/\alpha }}} \right)}^2}}}$$

9 $$\frac{{dR}}{{dp}} = \frac{{{R_f} - {R_i}}}{\alpha }\frac{{{e^{\left( {p - {p_0}} \right)/\alpha }}}}{{{{\left( {1 + {e^{\left( {p - {p_0}} \right)/\alpha }}} \right)}^2}}}$$

**Figure 11:**
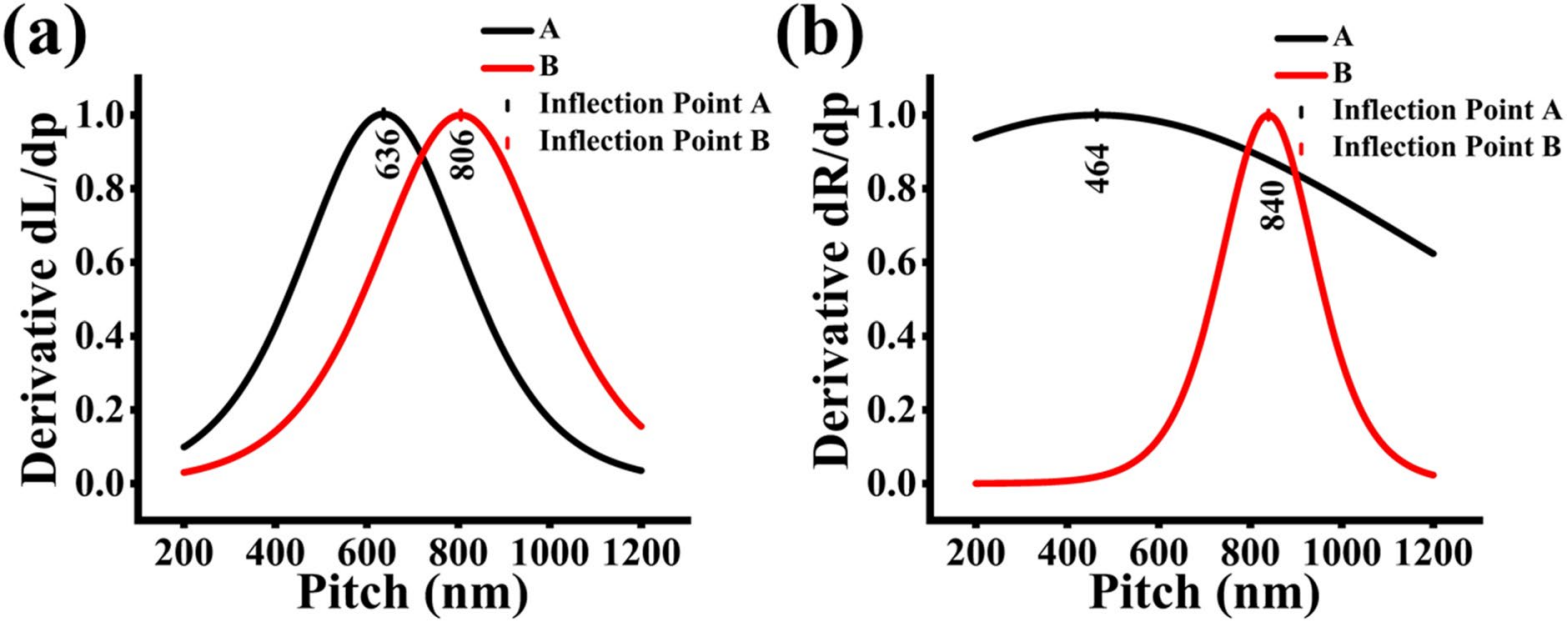
The first derivative of Boltzmann sigmoidal fit results for sample A and sample B for (**a**) length and (**b**) radial extension, respectively.

The first derivative relates to the modeling profile for the incremental change in length and radial extension in terms of the variation of the pitch length as opposed to incremental change in the deposition given by Sharma et al.^10^ The normalized plots of $\frac{dL}{dp}$ and $\frac{dR}{dp}$ are given in the following figures. The inflection point is graphically indicated by the functions’ maximum in Fig. 11a,b. The inflection point for patterned growth then represents the value where the total flux has the maximum effect of axial and radial growth rates. Furthermore, the pitch-dependent effects of the flux on the axial and radial growth rates begin to lessen as the pitch length increases beyond the extracted inflection point.

### Relationship to growth environment parameters

Inference to physical parameters can be made from the extracted sigmoidal parameters given by the model. Minimum and maximum growth rates for a given growth environment are extracted and related by the slope, *α*. Smaller values of *α* indicate quicker transition from the minimum to maximum growth rates. The sigmoidal fitting extracted similar values for *α* for the axial growth in both nitride samples. This result suggests that this parameter is less time-dependent and more dependent upon the growth conditions, i.e. the group III flux, group V fluxes, substrate temperature, etc., for the axial growth in the patterned NWs. Additionally, the slope values extracted for the radial growth are not similar in samples A and B. This suggests that there is a more prominent time dependence contributing to the radial growth rate slope in addition to the growth environment parameters.

Furthermore, the size of *α* indicates the range of pitch lengths, where the secondary fluxes influence the incremental changes in length and radial extension. The values of *α* can be associated with the FWHM of the functions, with smaller values of *α* corresponding to narrower FWHM in the $\frac{dL}{dp}$ and $\frac{dR}{dp}$ functions, as observed in both samples in Fig. 11a, in sample B in Fig. 11b, and vice -versa. The correlation to the FWHM of the given functions to pitch dependency is that narrower FWHM indicates the range of pitch lengths where the secondary fluxes make a significant contribution to the incremental change of the length and radial extension. Outside of this range, the secondary flux contribution is minimal. This observation further corroborates the suppressive effect of the presence of nitrogen on the secondary fluxes at lower pitch lengths and the dominating contribution of the primary flux over the diminishing contributions of the secondary fluxes at higher pitch lengths. Additionally, the range of pitch lengths associated with the value of *α* is consistent with PL analysis, which also exhibits the overlap of GaAsSb and dilute nitride related spectra in this pitch length region. Furthermore, the inflection point also falls in this range.

The analysis of our data shows the pitch influences the direct impinging flux and secondary fluxes, thus affecting the axial and radial growth rates, which can be correlated to Sb and N incorporation. The higher Sb content composition stem which resulted in bent and non-vertical NWs in Sample A, was circumvented with a low Sb stem grown on top of a shorter GaAs stem in Sample B, improving the vertical yield. Though multiple NWs were observed to nucleate out of a single patterned hole in sample B, future work in patterned hole optimization can resolve this issue and further improve results.

N incorporation improves the bandgap tuning over a wider wavelength range in comparison to non-nitride patterned NWs, as ascertained in the PL spectra. The lower pitch length of 200 nm is dominated by GaAsSb PL spectra, while for the intermediate pitch lengths between 400 and 800 nm, broad PL spectra consist of overlapping peaks associated with Sb and N incorporation in both the samples. Furthermore, for the intermediate pitch length region, the PL spectra are found to be more symmetric and broader due to the increased N contribution. It is to be noted that no post growth annealing has been done, which is commonly required for a dilute nitride system to annihilate the N-related defects, and also, the NWs have not been passivated, which leaves room for significant improvement in PL response in future work.

Additionally, the logistic sigmoidal fitting model proved to be very accurate in capturing both the axial and radial growth rate trends in patterned dilute nitride GaAsSbN NW samples. The extracted inflection points, *p*_*0*_, of 636 nm and 806 nm, for axial growth rate correlate very well with the homogeneous broad peaks revealed by PL analysis at these pitch lengths. Furthermore, the inflection point indicates the pitch length where the pitch dependent effects on the secondary fluxes play a prominent role in axial and radial growth of patterned NWs. Additionally, the values of the sigmoidal slope, *α*, correlate well with the range of pitches where Sb and N incorporation are observed with broad PL spectra. This clearly demonstrates the sigmoidal model as a powerful modeling tool in providing an initial guideline in designing a patterned array of optimized pitch length range that can be extended to a class of highly mismatched alloy compounds, as in dilute nitrides, without the need for PL analysis at every pitch length.

## Conclusion

Optimized processing and growth conditions for the best coverage of the patterned holes were achieved for 200 nm pitch length. The first report of dilute nitride patterned array shows bandgap tuning over a large wavelength range of ~ 75 meV over a pitch length range from 200 to 1200 nm, which is greater than the reported tuning of ~ 40–50 meV in patterned non-nitride GaAsSb NWs. PL data reveals symmetric and broad peaks consisting of overlapping GaAsSb and GaAsSbN PL spectra within the intermediate pitch length range from 400 to 800 nm. Additionally, the first reports of the application of the sigmoidal fitting model show excellent agreement with axial and radial growth of patterned NWs. The extracted parameters of inflection point, and slope can be used as a rubric for optimal pitch length for patterned array design of homogeneous nitride NWs, thus eliminating the use of detailed PL analysis.

## Supplementary Information

Supplementary Information

## References

[1] Dai X, Zhang S, Wang Z, Adamo G, Liu H, Huang Y, Couteau C, Soci C (2014). GaAs/AlGaAs nanowire photodetector. Nano Lett..

[2] Sharma M, Ahmad E, Dev D, Li J, Reynolds CL, Liu Y, Iyer S (2019). Improved performance of GaAsSb/AlGaAs nanowire ensemble Schottky barrier based photodetector via in situ annealing. Nanotechnology.

[3] Aiello A, Hoque AKMH, Baten MZ, Bhattacharya P (2019). High-gain silicon-based InGaN/GaN dot-in-nanowire array photodetector. ACS Photon..

[4] Arefinia Z, Asgari A (2015). Optical and electrical modeling of solar cells based on graphene/Si nanowires with radial p-i-n junctions. Sol. Energy Mater. Sol. Cells.

[5] Parakh M, Johnson S, Pokharel R, Ramaswamy P, Nalamati S, Li J, Iyer S (2020). Space charge limited conduction mechanism in GaAsSb nanowires and the effect of in situ annealing in ultra-high vacuum. Nanotechnology.

[6] Prithviraj D, Manish S, Surya N, Lewis Reynolds J, Yang L, Shanthi I (2018). Molecular beam epitaxial growth of high quality Ga-catalyzed GaAs1–x Sb x (x > 0.8) nanowires on Si (111) with photoluminescence emission reaching 1.7 μm. Semicond. Sci. Technol..

[7] Nalamati S, Sharma M, Deshmukh P, Kronz J, Lavelle R, Snyder D, Reynolds CL, Liu Y, Iyer S (2019). A study of GaAs 1–x Sb x axial nanowires grown on monolayer graphene by Ga-assisted molecular beam epitaxy for flexible near-infrared photodetectors. ACS Appl. Nano Mater..

[8] Tomioka K, Ikejiri K, Tanaka T, Motohisa J, Hara S, Hiruma K, Fukui T (2011). Selective-area growth of III–V nanowires and their applications. J. Mater. Res..

[9] Vukajlovic-Plestina J, Kim W, Ghisalberti L, Varnavides G, Tütüncuoglu G, Potts H, Friedl M, Güniat L, Carter WC, Dubrovskii VG, Fontcuberta MA (2019). Fundamental aspects to localize self-catalyzed III–V nanowires on silicon. Nat. Commun..

[10] Sharma M, Karim MR, Kasanaboina P, Li J, Iyer S (2017). Pitch-induced bandgap tuning in self-catalyzed growth of patterned GaAsSb axial and GaAs/GaAsSb core-shell nanowires using molecular beam epitaxy. Cryst. Growth Des..

[11] Kruse JE, Lymperakis L, Eftychis S, Adikimenakis A, Doundoulakis G, Tsagaraki K, Androulidaki M, Olziersky A, Dimitrakis P, Ioannou-Sougleridis V, Normand P, Koukoula T, Kehagias T, Komninou P, Konstantinidis G, Georgakilas A (2016). Selective-area growth of GaN nanowires on SiO2-masked Si (111) substrates by molecular beam epitaxy. J. Appl. Phys..

[12] Hetzl M, Kraut M, Winnerl J, Francaviglia L, Döblinger M, Matich S, Fontcuberta Morral A, Stutzmann M (2016). Strain-induced band gap engineering in selectively grown GaN-(Al, Ga)N core-shell nanowire heterostructures. Nano Lett..

[13] Bharatan S, Iyer S, Nunna K, Collis WJ, Matney K, Reppert J, Rao AM, Kent PRC (2007). The effects of annealing on the structural, optical, and vibrational properties of lattice-matched GaAsSbN/GaAs grown by molecular beam epitaxy. J. Appl. Phys..

[14] Nunna K, Iyer S, Wu L, Li J, Bharatan S, Wei X, Senger RT, Bajaj KK (2007). Nitrogen incorporation and optical studies of GaAsSbN/GaAs single quantum well heterostructures. J. Appl. Phys..

[15] Patra NC, Bharatan S, Li J, Tilton M, Iyer S (2012). Molecular beam epitaxial growth and characterization of InSb1 − xNx on GaAs for long wavelength infrared applications. J. Appl. Phys..

[16] Devkota S, Parakh M, Johnson S, Ramaswamy P, Lowe M, Penn A, Reynolds L, Iyer S (2020). A study of n-doping in self-catalyzed GaAsSb nanowires using GaTe dopant source and ensemble nanowire near-infrared photodetector. Nanotechnology.

[17] Nalamati S, Devkota S, Li J, Lavelle R, Huet B, Snyder D, Penn A, Garcia R, Reynolds L, Iyer S (2020). Hybrid GaAsSb/GaAs heterostructure core-shell nanowire/graphene and photodetector applications. ACS Appl. Electron. Mater..

[18] Pokharel R, Ramaswamy P, Devkota S, Parakh M, Dawkins K, Penn A, Cabral M, Reynolds L, Iyer S (2020). Epitaxial high-yield intrinsic and Te-doped dilute nitride GaAsSbN nanowire heterostructure and ensemble photodetector application. ACS Appl. Electron. Mater..

[19] Prithviraj D, Jia L, Surya N, Manish S, Shanthi I (2019). Molecular beam epitaxial growth of GaAsSb/GaAsSbN/GaAlAs core-multishell nanowires for near-infrared applications. Nanotechnology.

[20] Yip S, Shen L, Ho JC (2019). Recent advances in III-Sb nanowires: From synthesis to applications. Nanotechnology.

[21] Li J, Iyer S, Bharatan S, Wu L, Nunna K, Collis W, Bajaj KK, Matney K (2005). Annealing effects on the temperature dependence of photoluminescence characteristics of GaAsSbN single-quantum wells. J. Appl. Phys..

[22] Wu L, Iyer S, Nunna K, Li J, Bharatan S, Collis W, Matney K (2005). MBE growth and properties of GaAsSbN/GaAs single quantum wells. J. Cryst. Growth.

[23] Iyer S, Wu L, Li J, Potoczny S, Matney K, Kent PRC (2007). Effects of N incorporation on the structural and photoluminescence characteristics of GaSbN/GaSb single quantum wells. J. Appl. Phys..

[24] Bharatan S, Iyer S, Matney K, Collis WJ, Nunna K, Li J, Wu L, McGuire K, McNeil LE (2006). Growth and properties of lattice matched GaAsSbN epilayer on GaAs for solar cell applications. Mater. Res. Soc. Symp. Proc..

[25] Braza V, Reyes DF, Gonzalo A, Utrilla AD, Ben T, Ulloa JM, González D (2017). Sb and N incorporation interplay in GaAsSbN/GaAs epilayers near lattice-matching condition for 1.0–1.16-eV photonic applications. Nanosc. Res. Lett..

[26] Xu Z, Saadsaoud N, Loke WK, Tan KH, Wicaksono S, Yoon SF, Lecoustre G, Decoster D, Chazelas J (2011). High-performance GaNAsSb/GaAs 155-{m waveguide photodetector. IEEE Trans. Electron. Dev..

[27] Kasanaboina PK, Li J, Iyer S, Ahmad E, Reynolds CL, Liu Y (2015). Self-catalyzed growth of dilute nitride GaAs/GaAsSbN/GaAs core-shell nanowires by molecular beam epitaxy. Appl. Phys. Lett..

[28] Kasanaboina P, Sharma M, Deshmukh P, Reynolds CL, Liu Y, Iyer S (2016). Effects of annealing on GaAs/GaAsSbN/GaAs core-multi-shell nanowires. Nanoscale Res. Lett..

[29] Manish S, Prithviraj D, Pavan K, Lewis Reynolds J, Yang L, Shanthi I (2017). Growth of defect-free GaAsSbN axial nanowires via self-catalyzed molecular beam epitaxy. Semicond. Sci. Technol..

[30] Pavan Kumar K, Sai Krishna O, Shifat Us S, Lewis Reynolds J, Yang L, Shanthi I (2015). Bandgap tuning of GaAs/GaAsSb core-shell nanowires grown by molecular beam epitaxy. Semicond. Sci. Technol..

[31] Sandra JG, Ray RL (2014). Model of patterned self-assisted nanowire growth. Nanotechnology.

[32] Chen A, Chua SJ, Chen P, Chen XY, Jian LK (2006). Fabrication of sub-100 nm patterns in SiO2 templates by electron-beam lithography for the growth of periodic III–V semiconductor nanostructures. Nanotechnology.

[33] Sharma, M., Iyer, S., Kasanaboina, P., Nanoengineering: Fabrication, P. O., Devices, X. I. V. Impact of processing and growth conditions on the site-catalyzed patterned growth of GaAs nanowires by molecular beam epitaxy. *Proc. SPIE Int. Soc. Opt. Eng.**10354* (2017).

[34] Navarro-Verdugo AL, Goycoolea FM, Romero-Meléndez G, Higuera-Ciapara I, Argüelles-Monal W (2011). A modified Boltzmann sigmoidal model for the phase transition of smart gels. Soft Matter.

[35] Frazier A, Wang L (2013). Modeling landscape structure response across a gradient of land cover intensity. Landsc. Ecol..

[36] Love, B., Revisiting Boltzmann kinetics in applied rheology. *SPE, Plast. Res. Online Doi* **2009,** *10*.

[37] Luna E, Guzmán A, Trampert A, Alvarez G (2012). Critical role of two-dimensional island-mediated growth on the formation of semiconductor heterointerfaces. Phys. Rev. Lett..

[38] Jing L, Luna E, Toshihiro A, Steenbergen EH, Yong-Hang Z, Smith DJ (2016). Evaluation of antimony segregation in InAs/InAs1–xSbx type-II superlattices grown by molecular beam epitaxy. J. Appl. Phys..

[39] Resendiz-Munoz J, Corona-Rivera MA, Ovando-Medina VM, Fernandez-Munoz JL, Zapata-Torres M, Marquez-Herrera A (2017). Mathematical model of Boltzmann's sigmoidal equation applicable to the set-up of the RF-magnetron co-sputtering in thin films deposition of BaxSr1-xTiO3. Bull. Mater. Sci..

[40] Robson MT, Dubrovskii VG, LaPierre RR (2015). Conditions for high yield of selective-area epitaxy InAs nanowires on SiO x /Si(111) substrates. Nanotechnology.

[41] Ahmad E, Karim MR, Hafiz SB, Reynolds CL, Liu Y, Iyer S (2017). A two-step growth pathway for high Sb incorporation in GaAsSb nanowires in the telecommunication wavelength range. Sci. Rep..

[42] De Jong E, LaPierre RR, Wen JZ (2009). Detailed modeling of the epitaxial growth of GaAs nanowires. Nanotechnology.

[43] Xu L, Huang Q (2014). Growth process modeling of III-V nanowire synthesis via selective area metal-organic chemical vapor deposition. IEEE Trans. Nanotechnol..

[44] Sokolovskii AS, Robson MT, LaPierre RR, Dubrovskii VG (2019). Modeling selective-area growth of InAsSb nanowires. Nanotechnology.

[45] Kelrich A, Calahorra Y, Greenberg Y, Gavrilov A, Cohen S, Ritter D (2013). Shadowing and mask opening effects during selective-area vapor-liquid-solid growth of InP nanowires by metalorganic molecular beam epitaxy. Nanotechnology.

[46] Sapteka AANG, Narottama AANM, Winarta A, Amerta Yasa K, Priambodo PS, Putra N (2018). Modelling of electric characteristics of 150-watt peak solar panel using Boltzmann sigmoid function under various temperature and irradiance. J. Phys: Conf. Ser..

[47] Luna E, Ishikawa F, Batista PD, Trampert A (2008). Indium distribution at the interfaces of (Ga, In)(N, As)∕GaAs quantum wells. Appl. Phys. Lett..

[48] Hulko O, Thompson DA, Simmons JG (2008). Comparison of quantum well interdiffusion on group III, group V, and combined groups III and V sublattices in GaAs-based structures. IEEE J. Selected Topics Quant. Electron..

[49] Dimo K (2006). Dependence of the growth rate of nanowires on the nanowire diameter. Crystal Growth Design.

