# Supplementary material for: Study of patterned GaAsSbN nanowires using sigmoidal model
Source: Sci Rep. 2021 Feb 25;11:4651. doi: 10.1038/s41598-021-83973-9 (PMC7907112; doi:10.1038/s41598-021-83973-9)
Supplement: Supplementary file 1 — Supplementary Information [file 41598_2021_83973_MOESM1_ESM.pdf]

Supplementary Information

Study of Patterned GaAsSbN Nanowires using Sigmoidal Model

Sean Johnson [1], **Rabin Pokharel**[2], **Michael Lowe**[1], **Hirandeep Kuchoor**[2], **Surya Nalamati**[1], **Klinton Davis**[3], **Hemali Rathnayake**[3], **and Shanthi Iyer**[2*]

[1] Department of Electrical and Computer Engineering, North Carolina A&T State University, Greensboro, North Carolina 27411, USA.

[2] Nanoengineering, Joint School of Nanoscience and Nanoengineering, North Carolina A&T State University, Greensboro, North Carolina 27401, USA.

[3] Nanoscience, Joint School of Nanoscience and Nanoengineering, University of North Carolina at Greensboro, Greensboro, North Carolina 27401, USA.

*Corresponding author email: iyer@ncat.edu.

S1. Comparison of non-patterned and patterned GaAsSbN NWs

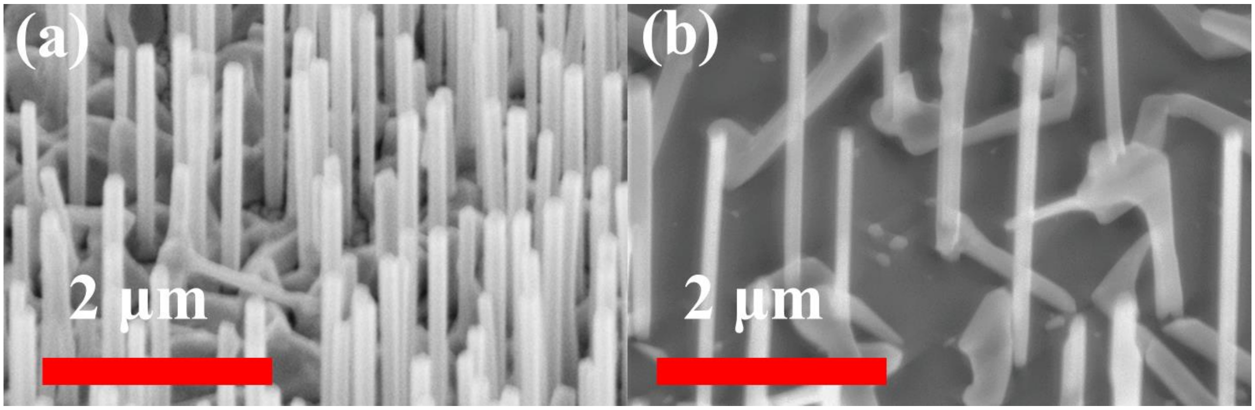

Supplementary Figure S1. SEM comparison of (a) non-patterned and (b) patterned GaAsSbN NWs.

Fig. S1a-b captures the differences observed in non-patterned and patterned GaAsSbN NW growth. Significant 2D deposition is observed in the non-patterned sample, which results in randomized nucleation of vertical NWs. Further optimization of the patterned hole diameter and Ga pre-deposition will reduce non-vertical NW orientation and increase coverage of the pattern holes during the growth.

S2. Comparison of GaAsSb and GaAsSbN PL spectra

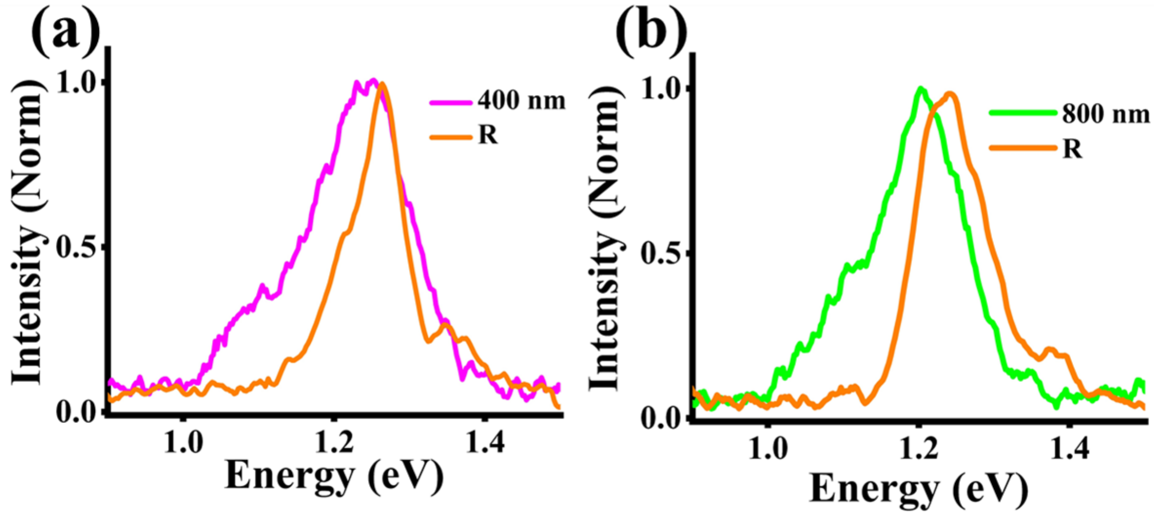

Supplementary Figure S2. Comparison of GaAsSb and GaAsSbN PL spectra at selected pitch lengths of (a) 400 nm and (b) 800 nm. R designates the GaAsSb reference sample at the respective pitch length.

Shown in Fig. S2a-b is the comparison of non-nitride and nitride PL spectra for patterned NWs. N incorporation is observed by the broadening of the spectra at on the low-energy side and red-shift

in the peak energy at the respective pitch lengths in comparison to the non-nitride reference spectra, designated as R.
